# Cardiac MR feature tracking identifies abnormal biventricular global strain values in biopsy-proven non-ischemic cardiomyopathies

**DOI:** 10.1186/1532-429X-17-S1-Q8

**Published:** 2015-02-03

**Authors:** Jeremy D Collins, Marcos Botelho, Madeline Stark, Daniel C Lee, Kevin Kalisz, Peter M Smith, Bradley D Allen, Maria L Carr, Bruce S Spottiswoode, James C Carr, Benjamin H Freed

**Affiliations:** 1 Radiology, Northwestern University, Chicago, IL, USA; 2 Feinberg School of Medicine, Northwestern University, Chicago, IL, USA; 3 Radiology, Case Western Reserve University, Cleveland, OH, USA; 4 Cardiology, Northwestern University, Chicago, IL, USA; 5 Cardiovascular MR Research & Development, Siemens Healthcare, Chicago, IL, USA

## Background

Cardiac MR is the reference standard for assessing biventricular systolic function and delayed enhancement. However, changes in systolic function as assessed by global parameters such as ventricular ejection fraction are late indicators of adverse structure-function changes. Myocardial strain has shown promise to identify abnormal potentially reversible regional myocardial function, preceding changes in the ejection fraction. Feature tracking cardiac MR is a novel technique to quantitate myocardial function, tracking biventricular myocardial features to generate Lagrangian strain values from existing cine balanced steady state free precession (bSSFP) data. The purpose of this study is to assess the feasibility of retrospective strain analysis in a patient cohort with biopsy-proven non-ischemic cardiomyopathy, comparing to strain data in healthy volunteers.

## Methods

26 consecutive patients (21 men, avg age 64 years) with a biopsy proven non-ischemic cardiomyopathy (NICM) and 20 healthy volunteers (14 men, avg age 44 years) underwent cardiac MR (CMR) imaging at 1.5T with bSSFP segmented cine imaging with a temporal resolution of 25-40 msec. bSSFP cine images were evaluated by a single observer using prototype software employing deformation field analysis to generate Lagrangian strain values for the left (LV - radial, circumferential, longitudinal) and right ventricles (RV - longitudinal). LV (LVEF) and RV ejection fraction (RVEF) were quantified on a dedicated workstation by a single observer. A two-tailed student's t-test assessed differences in strain values between groups, with an alpha of 0.05 chosen to demarcate statistical significance.

## Results

Retrospective strain analysis was successful in all subjects. Mean LVEF and RVEF for the patient cohort was 41.2% and 35.9%, respectively, whereas all volunteers demonstrated LVEF and RVEF ≥ 55% and 45%, respectively. Mean (SD) global strain values for patients and [volunteers] were: LV longitudinal -10.8 (3.9) [-15.5 (2.5)]; LV circumferential -10.9 (4.1) [-17.2 (1.9)]; LV radial 20.1 (11.4) [36.2 (8.6)]; RV longitudinal -3.7 (6.4) [-18.3 (3.5)]. All strain parameters were significantly different between patients and volunteers (p<0.001). In 7 patients with LVEF>50%, LV strain parameters were also significantly different from volunteers, (p<0.007).

## Conclusions

This feasibility study demonstrated statistically significant differences in retrospectively derived biventricular myocardial strain parameters between patients with biopsy proven NICM and healthy volunteers. Importantly, LV strain parameters in patients with preserved LVEF were significantly different from volunteers. Myocardial strain analysis can be performed using feature tracking without a dedicated strain acquisition. Additional studies are warranted to validate our results, assess the magnitude of myocardial strain changes with age, and explore the potential of this technique to identify early functional changes preceding irreversible myocardial injury in NICM.

## Funding

N/A.

**Figure 1:**
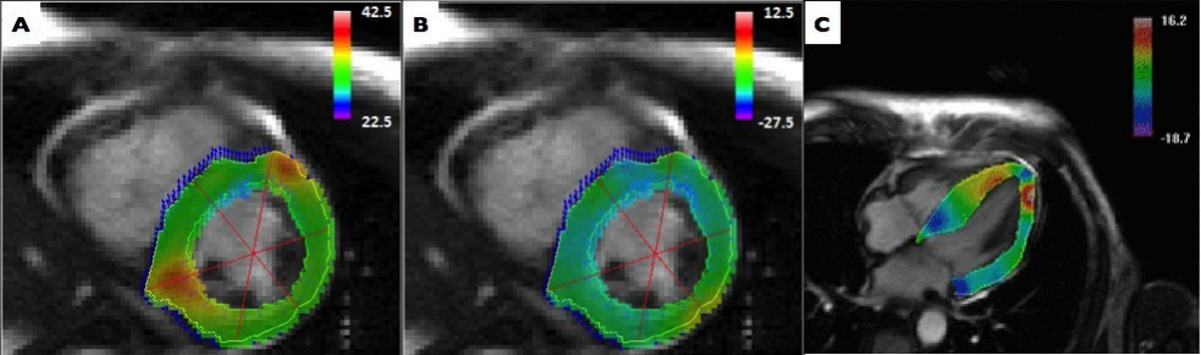
Representative visualization of peak systolic left ventricular radial (A), circumferential (B), and longitudinal (C) Lagrangian strain analysis.

